# Exploring wellbeing in first year medical students amidst a curriculum change

**DOI:** 10.1186/s12909-021-02678-9

**Published:** 2021-05-01

**Authors:** Deborah Merrick, Yvonne Mbaki, Margaret K. Pratten, Timothy G. Simpson

**Affiliations:** School of Life Sciences, Faculty of Medicine & Health Sciences, University of Nottingham, Medical School, Queen’s Medical Centre, Nottingham, NG7 2UH UK

**Keywords:** Undergraduate medical students, Wellbeing, Curriculum change

## Abstract

**Background:**

The support of student wellbeing features highly in all higher education institutional agendas. For medical students good physical and mental health can help prevent burnout, equip students for their future healthcare setting and indirectly improve patient care. At the University of Nottingham (UK), we were keen to explore undergraduate medical students perceived wellbeing before, during, and after an early years’ (years 1-3) curriculum change. A restructure of the curriculum enabled personal wellbeing sessions to be embedded and directly linked to the pastoral support system.

**Methods:**

Students’ perceived wellbeing was assessed through a questionnaire distributed to three cohorts of first year students at the start and end of the autumn semester.

**Results:**

The data showed a clear improvement of perceived physical health at the end of the first semester following the curriculum change, alongside increased mood and ability to relax. A surprising outcome of this study was that students reported increased stress levels at the end of the semester, which we believe may be attributed to the change in assessment within the new curriculum. Our medical students are now facing end of year summative examinations, but are acutely aware of their progress as they undertake frequent formative assessments during the year. We propose that comparison of performance with peers is having a direct impact on perceived stress in these cohorts.

**Conclusions:**

The study has shown that embedding wellbeing in the curriculum can have positive effects even within a changing curriculum. The importance of evolving wellbeing provision and support based on the needs of the student population is essential and probably never more in need than at this moment in time.

**Supplementary Information:**

The online version contains supplementary material available at 10.1186/s12909-021-02678-9.

## Background

Wellbeing as a psychological consideration took on new importance during the 1970’s and is now a regular consideration in many pedagogical studies [1]. Definitions are varied with some authors considering subjective wellbeing an evaluation of life in positive terms (‘life satisfaction’) which ‘*relies on the standards of the respondent to determine what is the good life*’ [1, 2]. As noted by Diener, many authors’ understanding mimics usage by non-philosophers, for example the idea of more ‘positive affect than negative affect’ [1, 3]. One such wellbeing model by Dodge et al., is suggested as the ‘*balance point between an individual’s resource pool and the challenges faced*’ [2]. Using a ‘see-saw’ representation, Dodge et al., suggested their model could ideally encapsulate the ability of a person to return to a state of wellbeing under different conditions. We, the authors, concur with this readily applicable model.

Fluctuations in an individual’s wellbeing are a common phenomenon throughout life. However certain stressful situations, such as transitioning from further to higher education, can impact negatively on a student’s wellbeing. This can include a loss of ‘decision-making’ guidance from parents, and the initial (potentially great) encounter with alcohol, coupled with the ‘impostor phenomenon’ [4–6]. Students on medical courses are known to suffer significant stressors - some possibly unique to their position [7] (and studies there-in). These can be classified as academic or non-academic [7]. Courses are protracted, with long teaching hours and terms. Fear of failure in a medical career and limited time for social relaxation or relationship forming are commonly problematic [6]. Additionally, there are the first encounters with human gross anatomy that some students find emotionally distressing, especially in the context of how to behave in front of peers [8]. Later years, such as those initial clinical placements, add patient encounters and real-world trauma to the mix. Studies have shown that stressors manifest as poor scholastic performance or more worryingly as unhealthy conditions including anxiety and depression [7]. Varyingly high levels of mental distress amongst medical students in a variety of different education systems have been noted [9, 10] in [7], often at a rate higher than aged matched populations [11] (and studies there-in) [6]. Unfortunately, in some cases excess stressors can lead to burnout, which not only has a detrimental effect on the individual, but can directly impact on patient care [11].

In their seminal paper of 2005, Dyrbye et al., stated that medical schools are charged with ensuring their graduates are professional [12]. As with other medical schools, the University of Nottingham (UoN) utilises a varied mix of didactic lectures, small group orientated case-based learning, practicals, and hands-on patient contact to ensure proficiency in a student’s chosen career. However, UoN also regularly revises its teaching approach, thereby ensuring a student’s continued efficacy within today’s healthcare setting. During the most recent reappraisal of UoN’s Bachelor of Medical Sciences (BMedSci) programme, an attempt to support student wellbeing was manifested through the implementation of a robust framework of support services and an objective realignment of curriculum delivery. Wellbeing interventions introduced cover study skills, nutrition, mental health, physical activity and goal setting and planning, delivered through a variety of approaches including one to one session and lectures (see supplementary table). Consequently, UoN’s new course layout features characteristics that optimise learning and yet contain a framework of student support strategies throughout. It is hoped this will enhance advantageous student coping mechanisms rather than non-advantageous mechanisms (e.g. social withdrawal, disengagement) [12]. Strategies are in place to improve wellbeing and as with other previously noted approaches, students are educated to notice personal imbalances [12]. Students are exposed to repeated high-stress/low stakes examinations in an attempt to aid adaptation and resilience [13]. As with other Schools, UoN medical students have increased contact with patients in the early years (preclinical phase) to motivate learning and to ease transitions [14] in [15] so reducing the ‘Shock of Practice’ noted by Boshuizen [16] in [17]. Additionally, a realignment of information presented in the early years of the BMedSci means a better understanding of basic science and an ability to link it to clinical reasoning.

Not only are such approaches ethical, but they also make sense in a time of increased marketization of universities that have led to a more ‘student-orientated’ provision of education [18]. With increased fees being encountered, students are keen to receive robust teaching and a valid learning experience [18–21]. As noted by Diener, measures of subjective wellbeing can be used to determine the efficacy of policy changes [22]. Using this rationale as a starting point, the authors decided to assess the robustness of the UoN’s medicine curriculum design and its effect on students by assessing their wellbeing during their first year on the course.

## Method

### Population selection

Three cohorts of first-year undergraduate students studying Medicine at the UoN between 2015 and 2020 were invited to participate in this study. The three cohorts represented students on the old curriculum (2015/16), the first year of the new curriculum (2017/18) and 2 years post curriculum change (2019/20). Student participation remained voluntary and anonymous at all times throughout the investigation. The study received ethical approval from the UoN School of Life Sciences Ethics Committee (No. B021019YM) throughout its duration.

### Questionnaire design

All participants were asked a number of questions relating to their perception of their own mental and physical wellbeing. Likert scale questions, commonly used in medical education [23], were used to establish personal attributes, relaxation methods and Perceived Stress Scale [24]. FANTASTIC (family, friends, activity, nutrition, toxins, alcohol, stress, sleep, personality type, insight and career) lifestyle assessment questions were also included and scored on a 3-point Likert scale from 0 (hardly ever), 1 (some of the time), to 2 (almost always) [25]. The reliability and validity of such an approach is reported elsewhere in the literature [26]. Students were asked to complete the questionnaire at the start of semester 1 shortly after commencing their studies (T1; October) and then again at the end of semester 1 (T2; December).

### Data analysis

Collected data were inputted into a Microsoft Excel spreadsheet for analysis and storage [27]. Quantitative Likert scale data were assigned numerical values ranging 1-5 or 0-2 for FANTASTIC lifestyle scoring. Percentages were calculated by (ordinal category response)/(total respondents for questionnaire) × 100; rounded to 1 decimal place. Percentages were also generated for aggregated ordinal categories (composed of options 1 + 2; 4 + 5). Aggregate option (1 + 2) was considered a ‘negative response’ to the question as posed. Aggregate option (4 + 5) was considered ‘positive’. Option 3 was considered ‘neutral’. Clustered column and diverged stack plots were formulated using percentages calculated as above and figures were rounded to two decimal places. Where appropriate for graphing purposes, categories deemed to represent negative responses were awarded a negative weighting.

## Results

First year UoN undergraduate medical students’ perception of their own wellbeing was assessed in three separate cohorts enrolled on the medicine course before (2015/16), during (2017/18) and after (2019/20) a curriculum change.

### Cohort response rate

High response rates were obtained for all year cohorts at each time point (see Table 1) thus it is believed the data is representative of the year group as a whole. Notably, T2 time points did display decreased responses compared to T1.

**Table 1:** Response rates of completed questionnaires for first year UoN medical students on the old curriculum (2015-16) and new-curriculum (2017-18 and 2019-20) at the beginning (T1) and end (T2) of the autumn semester

Cohort Year	T1 Response rate (*n*=)	T2 Response rate (*n*=)
2015/16	82.26% (209/253)	44.66% (113/253)
2017/18	89.45% (246/275)	48.36% (133/275)
2019/20	87.77% (245/296)	61.49% (182/296)

### Positive cohort observations/trends in the new curriculum

**Fig. 1:**
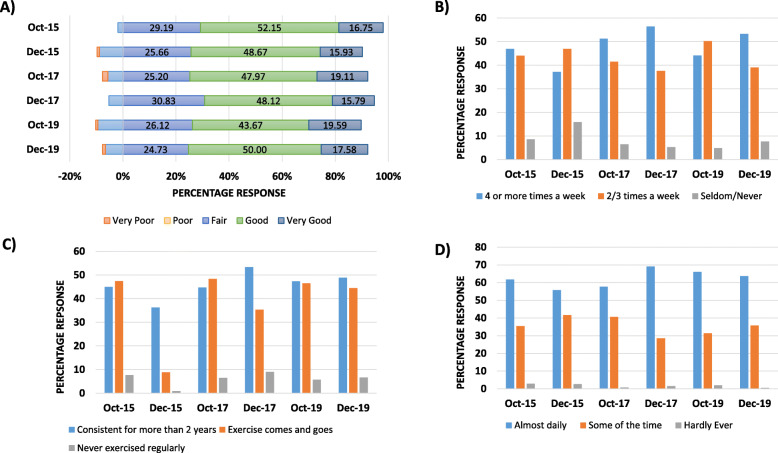
Positive trends observed in new curriculum: **a** Physical Health (negative responses awarded a negative rating); **b** Active Exercise – 30 min e.g. running, cycling, fast walk; **c** Maintaining Exercise over the long term, and **d** Relaxation and Enjoyment of leisure time

All first-year medical students were asked a series of questions related to their lifestyle choices and physical health. There was a distinct decrease in students’ perception of their own physical health at the end of the autumn semester on the old curriculum (1.91 to 9.73%; 4/209 to 11/113 aggregate for ‘very poor’ & ‘poor’ responses, Fig. 1a). This was in conjunction with a reduction in frequency of exercise (students reporting ability to exercise 4 times per week for 30 min or more, dropped from 46.89 to 37.17%; 98/209 to 42/113, Fig. 1b), and students’ ability to maintain regular exercise over the long term (ability to keep exercise levels consistent with 2 yearly average dropped from 44.98 to 36.28%; 94/209 to 41/113 aggregate, Fig. 1c). In contrast, students on the new curriculum reported a higher frequency of exercise as the semester progressed (T2 2017: 56.39% or 75/133; and T2 2019: 53.30% or 97/182 aggregate), and their ability to maintain exercise similarly increased (T2 2017: 53.38% or 71/133; and T2 2019: 48.90% or 89/182).

The ability to relax and overall enjoyment was seen to decrease between T1 and T2 on the old curriculum (with a drop of approximately 3 - 71.77% to 69.03%, 150/209 to 78/113). By 2019, this trend was reversed following the curriculum change, so that students at the end of the autumn semester now reported an increase in enjoyment and ability to relax (57.14 to 64.29%, 140/245 to 117/182, Fig. 1d).

### Negative cohort observations/trends in the new curriculum

**Fig. 2:**
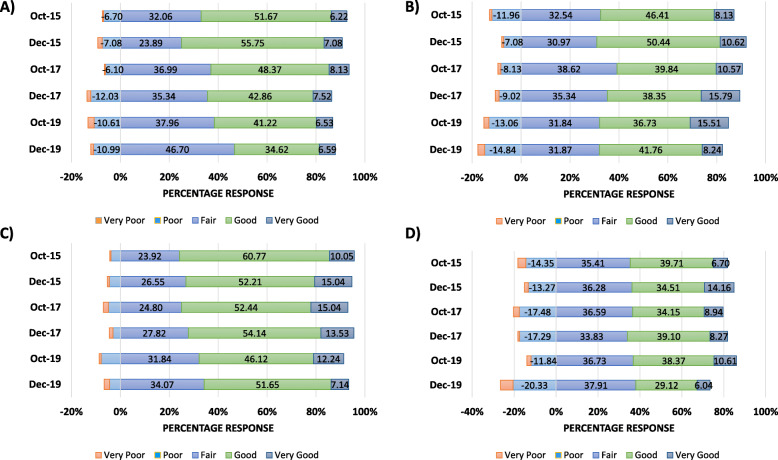
Negative trends observed in new curriculum **a** Ability to Manage Stress; **b** Ability to Keep Stress in Perspective; **c** Mood; and **d** General Levels of Energy. Negative responses awarded a negative rating

All first-year medical students were asked a series of questions related to their perceived stress level and their ability to manage stress. On the old curriculum students’ ability to manage stress was improved at the end of the semester compared to the beginning (with aggregate positive responses of ‘good’ and ‘very good’ being 57.89 to 62.83%, 121/209 to 71/113 aggregate at T1 and T2 respectively, Fig. 2a). This cohort of students also reported being more able to keep stress in perspective at the end of the semester (with aggregate positive responses of ‘good’ and ‘very good’ being 54.55 to 61.06%, 114/209 to 69/113, aggregate at T1 and T2 respectively, Fig. 2b).

However, following the introduction to the new curriculum, students reported being less able to manage stress at the end of the semester (with aggregate negative responses of ‘very poor’ and ‘poor’ increasing from 6.50 to 13.53%, 16/246 to 18/133, aggregate in 2017) or similar levels of inability in 2019: 13.06 to 12.09%, 32/245 to 22/182 in 2019). Additionally, their ability to keep this stress in perspective also decreased or remained determinedly negative (2017: 9.35 to 10.53%, 23/246 to 14/133; and 2019: 15.10 to 17.58%, 37/245 to 32/182).

Self-reporting of mood at the beginning and end of the first semester was comparable on the old (2015/16) and new (2017/18) curriculum (2015: 70.81 to 67.26%, 148/209 to 76/113; and 2017: 67.48 to 67.67%, 166/246 to 90/133 [positive response aggregate], Fig. 2c). General energy levels in these cohorts increased at the end of the semester (2015: 46.41 to 48.67%, 97/209 to 55/113; and 2017: 43.09 to 47.37%, or 106/246 to 63/133, Fig. 2d). However, in the 2019/20 cohort, mood, and general level of energy were much lower at the end of the semester - an observation not previously seen (e.g. negative response aggregate increasing from 13.88 to 26.37%, 34/245 to 48/182). These findings are also seen in conjunction with similar numbers of students reportedly feeling depressed at the end of the autumn semester whilst studying on the new curriculum (2019: 8.16 to 7.69%, 20/245 to 14/182).

First-year medical students on the old curriculum reported an increased ability to manage their time as the semester progressed. This positive trend was reversed following the introduction of the new curriculum, with students reporting a decrease in time management (negative responses: 2017: 11.38 to 15.04%, 28/246 to 20/133; and 2019: 15.10 to 20.33%, 37/245 and 37/182 aggregate).

### Cohort observations that have remained stable from old and new curriculum

First-year medical students reported their communication skills remained relatively unchanged from the start to end of the first semester in all cohorts irrespective of curriculum. Similarly, students’ ability to work in a team remained elevated at the start and end of the semester (all positive response aggregates in excess of 82%).

**Fig. 3:**
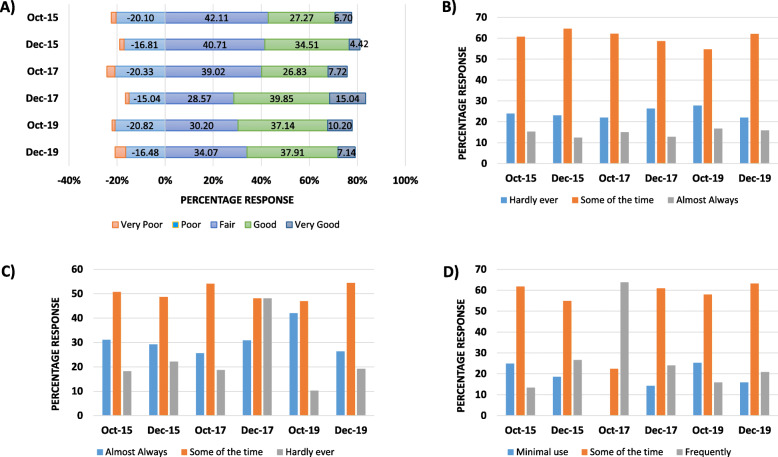
Comparable trends observed in new curriculum in relation to **a** Ability to Control Anxiety (negative response awarded a negative rating); **b** Anxiety/Worry Frequency; **c** Eat 5 or more servings of fruit and vegetables daily; a day; and **d** Excess Sugar, Salt, Animal Fats, or Junk Foods

Students’ self-reported ability to manage levels of anxiety fluctuated in different cohorts, with negative response levels generally dropping later in the semester (2015: 22.01 to 18.58% or 46/209 to 21/113; and 2017: 23.58 to 16.54%, or 58/246 to 22/133, Fig. 3a). Commonly, management of anxiety levels were more positive on the new curriculum (2015: 33.97 to 38.94%, or 71/209 to 44/113; 2017: 34.55 to 54.89% or 85/246 to 73/133; and 2019: 47.35 to 45.05%, 116/245 to 82/182). This trend was observed alongside a reported decrease in the frequency of anxiety at the end of the semester in all student cohorts (e.g. in 2017 by T2 26.32% or 35/133 up from 21.95% or 54/246, Fig. 3b).

A series of questions related to lifestyle choices identified that student’s healthy nutrition (specifically 5-a-day consumption) dropped in the 2015/16 and 2019/20 cohorts as the semester progressed (2015: 31.10 to 29.20% or 65/209 to 33/113, and 2019: 42.04 to 26.37%, 103/245 to 48/182, Fig. 3c). Interestingly, there was a peak in healthy nutrition in the 2017/18 cohort (T1: 25.61% or 63/246 to 30.83% or 41/133 at T2). Less healthy lifestyle choices including excess intake of sugar, salt, fat, junk food and alcohol fluctuated across student cohorts as they progressed from the beginning to the end of the autumn semester. In 2015, minimal use of these substances dropped from 24.88 to 18.58%, or 52/209 to 21/113 but in 2019 this response fell from 25.31 to 15.93% or 62/245 to 29/182 indicating a less healthy approach; Fig. 3d. Students self-reported body mass index (BMI) also decreased from start to the end of the semester on both the old and new curriculum. Interestingly, all student cohorts included in this study reported a comparable increased ability to problem solve as the semester progressed (2015: 74.64 to 84.96% or 156/209 to 96/113; 2017: 67.48 to 73.68% or 166/246 to 98/133, and 2019: 76.73 to 71.43% or 188/245 to 130/182).

## Discussion

It is the belief of many, including the authors of this study, that although students should rightly be prepared to cope within today’s healthcare setting, they need adequate support to acclimate during this process. Thus, it is the challenge of curriculum decision making to provide a supportive, yet wide enough context, for these various starting states. The change in the early years’ medical curriculum at UoN allowed us the opportunity to adopt a unique wellbeing approach that is fully embedded within the curriculum (see supplementary table for a summary of interventions). In year 1 of the new curriculum, the focus is to explore and help students question their own physical health, which we believe contributed to some positive and tangible lifestyle outcomes in our study, through the observed increment in awareness and frequency of physical activity. This shows a real attitudinal change which is something we plan to nurture and expand further by implementing an exciting Interprofessional Learning initiative based around physical health in the coming academic year. First year medical students also appear more positive in relation to their ability to relax, impacting overall mood at the end of the semester. One may postulate that this favourable outcome may well align to increased positive coping strategies, such as increased physical activity.

As with any well executed and planned curriculum changes one would anticipate many of the perceived wellbeing parameters to be unchanged e.g. communication and ability to problem solve increased throughout the semester in all cohorts irrespective of the curriculum. The spring semester in year 1 heralds the introduction of the case-based learning so we are keen to explore whether this approach has any influence on any of these important competencies [28]. Although cohorts report reasonable comparable levels of worry and anxiety throughout the semester, it is interesting to note that there appears to be emerging a more positive perception of students’ ability to manage their anxiety. We now direct our medical students to openly discuss and rate their own wellbeing (out of 10) during pastoral meetings with their personal tutors. We also place great emphasis on our students engaging in extracurricular activities when they start university which anecdotally comes as a surprise to our students. It is likely that with their being a great emphasis on academic achievement pre-university, the message of ‘it’s good for you (mental and physical wellbeing) to engage and make time to do these important activities’ is surprising to them. Although speculative, this encouragement may have gone some way to allow students to manage anxiety more positively, enabling them to keep things in perspective [29, 30].

UoN’s BMedSci programme encourages applications from high achieving individuals (possibly prone to perfectionism-based study approaches) and then places them in a cohort of equally intelligent/motivated students. Our results indicate that with the inclusion of a new curriculum our students are perceiving themselves to being more stressed at the end of the autumn semester, which was not observed in the old curriculum. As a surprising observation, it is likely that with summative examinations having been moved to the end of the spring semester (as opposed to end of autumn semester and end of spring semester in the old curriculum), students may be feeling the pressures of these high-stake examinations. Whilst the new curriculum provides students with the experience of formative examinations at regular intervals throughout the academic year (every 6 weeks, administered in a supervised timetabled session) to gauge their progress, it may be the case that students are placing great emphasis on their performance in the formatives, leading to the increased stress. Notably, whilst these students were perceived to be more stressed, they felt better able to cope at the end of the semester. Based on the findings in this study, the UoN Medical School is looking at ways to support our students with their approach and attitude towards their formative examinations. In 2020/21 we are launching a supportive collaborative engagement between staff and near-peer students, with the acknowledgment on the restrictions imposed from COVID-19 on face-to-face interactions. It is hoped that focussed student-led support groups will aid in the maintenance of cohort wellbeing as has been reported in other settings [12].

This study, like others of its type, may suffer the hidden impact of confounding elements that could lead to inferential ambiguity. The authors acknowledge that hidden factors, such as the educational history and demographics of the students, could influence results and mean a clear cause/effect relationship is hard to define. However, the protracted nature of the study coupled with its limited conclusions would potentially offer a counter-argument to significant bias.

### Limitations of the study

The authors are acutely aware that the reported findings may not be representative of other cohorts of medical students studying elsewhere in the UK or internationally. Indeed, our course provides a unique experience i.e., full body cadaveric dissection, as well as the award of two degrees (BMedSci & Bachelor of Medicine, Bachelor of Surgery, BMBS). Therefore, we acknowledge our data may not be easily extrapolated to other institutional settings. The data presented in this study does not allow comparison of individual student responses at the start and end of the first semester. Complete anonymity was chosen to ensure students completed the questionnaire as openly and honestly as possible, without a reticence to disclose information that students feel may come to harm their future career (should external agencies encounter the information). Thus, the analyses are cross-sectional comparisons and it is appreciated that the slightly reduced response rates (noted at T2) may have inadvertently skewed the data and the observations therein.

## Conclusion

As medical educators, we play a pivotal role in ensuring our students engage with activities that promote positive wellbeing, in preparation for their future professional careers. At UoN Medical School we are addressing through embedding wellbeing within the curriculum and pastoral support offered by personal tutors. We are keen to expand our provision to ensure we evolve to the needs of our students. We anticipate this is going to be ever more important for our future medical students as they enter higher education with Covid-19 pandemic restrictions imposed.

## Supplementary Information

**Additional file 1.**

**Additional file 2.**

## Data Availability

The datasets used and/or analysed during the current study are available from the corresponding author on reasonable request.

## Abbreviations

BMedSci: Bachelor of Medical Sciences
UK: United Kingdom
UoN: University of Nottingham
