# Supplementary material for: Exploring wellbeing in first year medical students amidst a curriculum change
Source: BMC Med Educ. 2021 May 1;21:252. doi: 10.1186/s12909-021-02678-9 (PMC8088313; doi:10.1186/s12909-021-02678-9)
Supplement: Supplementary file 1 — Additional file 1. [file 12909_2021_2678_MOESM1_ESM.docx]

**University of Nottingham School of Life Sciences Research & Ethics Committee approved**

**Title of Project:** *Promoting Wellbeing in Undergraduate Students*

**Lead Investigators: *Dr Margaret Pratten, Dr Yvonne Mbaki, Dr Deborah Merrick, Dr Tim Simpson***

*This questionnaire is part of a BMedSci Research study looking into the wellbeing of undergraduate and postgraduate students. Please fill out this questionnaire honestly. All results will remain anonymous.*

Please specify your course of study [Please select one that applies]

| A100 Undergraduate Entry Medicine |  |
| --- | --- |
| A101 Graduate Entry Medicine |  |
| A108 Medicine with a foundation year |  |
| A10L Undergraduate Entry Medicine |  |
| A18L Medicine with a foundation year |  |
| Biochemistry |  |
| Pharmacy |  |

What is your current year of study?

| Year 1 |  |
| --- | --- |
| Year 2 |  |
| Year 3 |  |
| Year 4 |  |
| Year 5 |  |

Are you a Home/EU or International Student? **Home/EU  International **

Please specify your ethnicity [Please tick one that applies]

| Asian or Asian British: |  Indian,   Pakistani,   Bangladeshi,   Chinese,   Any other Asian background |
| --- | --- |
| Black or Black British: |  Caribbean,   African,   Any other Black background |
| White: |  British,   Irish,   Any other White background |
| Mixed: |  White and Black Caribbean,   White and Black African,   White and Asian,   Any other mixed background |
| Other Ethnic Group (e.g. Arab): | Please specify: |
| Prefer not to say |  |

Please specify your gender

| Male | Female | Other | Prefer not to say |
| --- | --- | --- | --- |
|  |  |  |  |

Do you identify as part of the LGBT+ population? **Yes / No / Prefer not to say**

|  | **Very poor** | **Poor** | **Fair** | **Good** | **Very good** |
| --- | --- | --- | --- | --- | --- |
| Ability to manage stress |  |  |  |  |  |
| Ability to relax |  |  |  |  |  |
| Mood |  |  |  |  |  |
| Ability to control anxiety |  |  |  |  |  |
| Ability to keep stress in perspective |  |  |  |  |  |
| Physical health |  |  |  |  |  |
| General level of energy |  |  |  |  |  |
| Ability to communicate |  |  |  |  |  |
| Problem solving |  |  |  |  |  |
| Time management |  |  |  |  |  |
| Ability to focus on the present moment (e.g. in a lecture) |  |  |  |  |  |
| Ability to work well in a team |  |  |  |  |  |

How much do you currently employ the following?

|  | **Not at all** | **Rarely** | **Sometimes** | **Quite a lot** | **Always** |
| --- | --- | --- | --- | --- | --- |
| Meditation |  |  |  |  |  |
| Healthy nutrition |  |  |  |  |  |
| Exercise |  |  |  |  |  |
| Social support |  |  |  |  |  |
| ‘Spirituality’ |  |  |  |  |  |
| Mindfulness |  |  |  |  |  |
| Social media |  |  |  |  |  |

Which of the above do you find most effective at relieving stress? _______________________

Based on the past month rate yourself in each of the following areas:

|  | | 2 points | 1 points | No points | Your score |
| --- | --- | --- | --- | --- | --- |
| **F**amily & Friends | Communication with others is open honest and clear | Almost always | Some of the time | Hardly ever |  |
|  | I get the emotional support that I need | Almost always | Some of the time | Hardly ever |  |
| **A**ctivity | Active exercise – 30 minutes e.g. running cycling fast walk | 4 or more times a week | 2-3 times a week | Seldom or never |  |
|  | Relaxation and enjoyment of leisure time | Almost daily | Some of the time | Hardly ever |  |
|  | Maintain my exercise over the long term | Consistent for more than 2 years | Exercise comes and goes | Never exercised regularly |  |
| **N**utrition | Eat 5 or more servings of fruit and vegetables daily | Almost always | Some of the time | Hardly ever |  |
|  | Excess sugar, salt animal fats, or junk foods | Minimal use | Some of the time | Frequently |  |
|  | BMI | <25 | 25-30 | Over 30 |  |
| **T**obacco & Toxins | Frequency of smoking | Never | Social smoker | Smoke on daily basis |  |
|  | Coffee, tea, cola, energy drinks | Under 3 per week | 3 – 6 per week | 6 or more per week |  |
| **A**lcohol | Average intake per week  large glass of wine = 3 units  pint of cider/beer =2.3-2.8 units  single shot = 0.9-1.4 units | 10 or less units per week | 10-14 units per week | More than 14 units per week |  |
| **S**leep | 7-9 hours sound sleep per night | Almost always | Some of the time | Hardly ever |  |
| **S**tress | Tend to cope well with stress | Almost always | Most of the time | Some of |  |
|  | Major stressful events in past year | None | 1-2 | 3 or more |  |
|  | Positive thinker | Almost always | Some of the time | Hardly ever |  |
|  | Anxiety, worry | Hardly ever | Some of the time | Almost always |  |
|  | Depression | Hardly ever | Some of the time | Almost always |  |
| **T**ype of  Personality | Sense of time urgency:  Impatience, anger and hostility | Hardly ever | Some of the time | Almost always |  |
| **C**areer | Satisfied with experience of university course so far | Almost always | Some of the time | Hardly ever |  |
|  |  |  |  | **TOTAL/40** |  |

Please list any sports/ exercise you do regularly

Do you play sport competitively? Yes / No

Have you joined a sports team? Yes / No

If you stopped playing a sport after starting University, in which year of study did you stop and why?

The questions in this scale ask about your feelings and thoughts **during the last month.** Indicate by placing a cross in the box that response that best represents *how often* you felt or thought a certain way:

|  | **Never** | **Almost never** | **Some-times** | **Fairly often** | **Very often** |
| --- | --- | --- | --- | --- | --- |
| In the last month, how often have you been upset because of something that happened unexpectedly? |  |  |  |  |  |
| In the last month, how often did you practice Mindfulness? |  |  |  |  |  |
| In the last month, how often did you find yourself doing things without paying attention (running on “automatic pilot”) |  |  |  |  |  |
| In the last month, how often have you felt nervous and "stressed"? |  |  |  |  |  |
| In the last month, how often have you felt confident about your ability to handle your personal problems? |  |  |  |  |  |
| In the last month, how often did you find yourself preoccupied with future or past events |  |  |  |  |  |
| In the last month, how often have you been angered because of things outside your control? |  |  |  |  |  |
| In the last month, how often have you felt difficulties were piling up so high that you could not overcome them? |  |  |  |  |  |

Please give brief examples of what has caused you stress in the last month *(e.g. finance, workload, personal relationships)*:

|  |
| --- |

*Thank you for taking the time to complete this questionnaire.*
