# Supplementary material for: Exploring wellbeing in first year medical students amidst a curriculum change
Source: BMC Med Educ. 2021 May 1;21:252. doi: 10.1186/s12909-021-02678-9 (PMC8088313; doi:10.1186/s12909-021-02678-9)
Supplement: Supplementary file 2 — Additional file 2. [file 12909_2021_2678_MOESM2_ESM.docx]

| **Intervention** | **Mode of delivery** | **Strategy** |
| --- | --- | --- |
| Houses | Group activity | Acknowledging the existence of a correlation between sense of belonging and student wellbeing, with a potential effect on academic performance, students were assigned to one of four ‘houses’. As a collaborative effort between students and staff, house members identified social activities (e.g. Christmas movie night) as a way of promoting camaraderie within the group. |
| *Near-peer study skills | Lecture | Delivered via testimonials, this two hour student-led lecture titled ‘Learning Medicine’ showcased tips of how to approach studying medicine. Additionally, championed by the medical society (MedSoc), embedded within this session was the promotion of welfare services/activities available to students. |
| Nutrition | Lecture | As an interactive session, the focus was to get students to (1) reflect on their own eating habits (2) understand the importance of eating a balanced diet (3) appreciate the link between nutrition and mental health. |
| Mental Health First Aid | Online resource | This eLearning course focused on providing students with a brief understanding of mental health and common health mental issues, with considerations of how students could support their peers in need. |
| Physical activity | Lecture | Delivered by staff from the university’s sports village, the primary objective of this session was to reinforce the health benefits of physical activity to the students. |
| Reflection wheel for effective goal setting and planning | One-to-one session | As part of the tutorial system students were required to appraise and review their progress on the course, with considerations of attitudes, behaviour and general wellbeing. Reflective in nature, this process involved students rating their engagement within the domains of (1) academic progress and development (2) wellbeing (3) career preparation and planning (4) planning and organisation (5) clinical experiences and skill development (6) attendance and engagement (7) personal interests and hobbies (8) finance. |

Supplementary Table: Summary of interventions embedded into the Early Years (years 1 and 2) Medical Curriculum at University of Nottingham.
